# Haematospirillum jordaniae Nonpurulent Cellulitis and Bacteremia: A Case Report

**DOI:** 10.7759/cureus.73126

**Published:** 2024-11-06

**Authors:** Pradeep Kumar Mada

**Affiliations:** 1 Infectious Diseases, Comanche County Memorial Hospital, Lawton, USA

**Keywords:** fluoroquinolones, freshwater, gram-negative bacteremia, haematospirillum jordaniae, infectious cellulitis

## Abstract

*Haematospirillum jordaniae*, a rare gram-negative bacterium, is emerging as a potential pathogen causing cellulitis and bacteremia in immunocompromised individuals. We present a case of a 49-year-old male patient with no comorbidities who developed cellulitis and bacteremia due to *H. jordaniae*. This case highlights the importance of considering *H. jordaniae* as a potential etiology in cellulitis and bacteremia. This organism is associated with bullous cellulitis of the lower extremities and bacteremia usually followed by abrasions when in contact with freshwater.

## Introduction

Cellulitis is a common bacterial skin infection characterized by inflammation of the dermal and subcutaneous tissues. *Staphylococcus aureus* and *Streptococcus pyogenes* are usually cellulitis-associated pathogens, particularly in immunocompetent individuals [1]. However, in immunocompromised patients, cellulitis can be caused by a broader spectrum of bacteria, including rare and unusual species.* Haematospirillum jordaniae* is a gram-negative, spiral-shaped bacterium reported as a novel genus and species in 2016 by the Centers for Disease Control and Prevention [2]. Although infrequently reported, *H. jordaniae* has been implicated in cellulitis and bacteremia, particularly in patients with underlying hematologic malignancies or compromised immune systems [3]. *H. jordaniae *was formerly considered an environmental bacterium with limited pathogenicity, but rising isolates showed a potential emerging pathogen. All cases occurred in male patients, and the pathogen showed a preference for infecting lower extremity injuries [2].

## Case presentation

**Figure 1:**
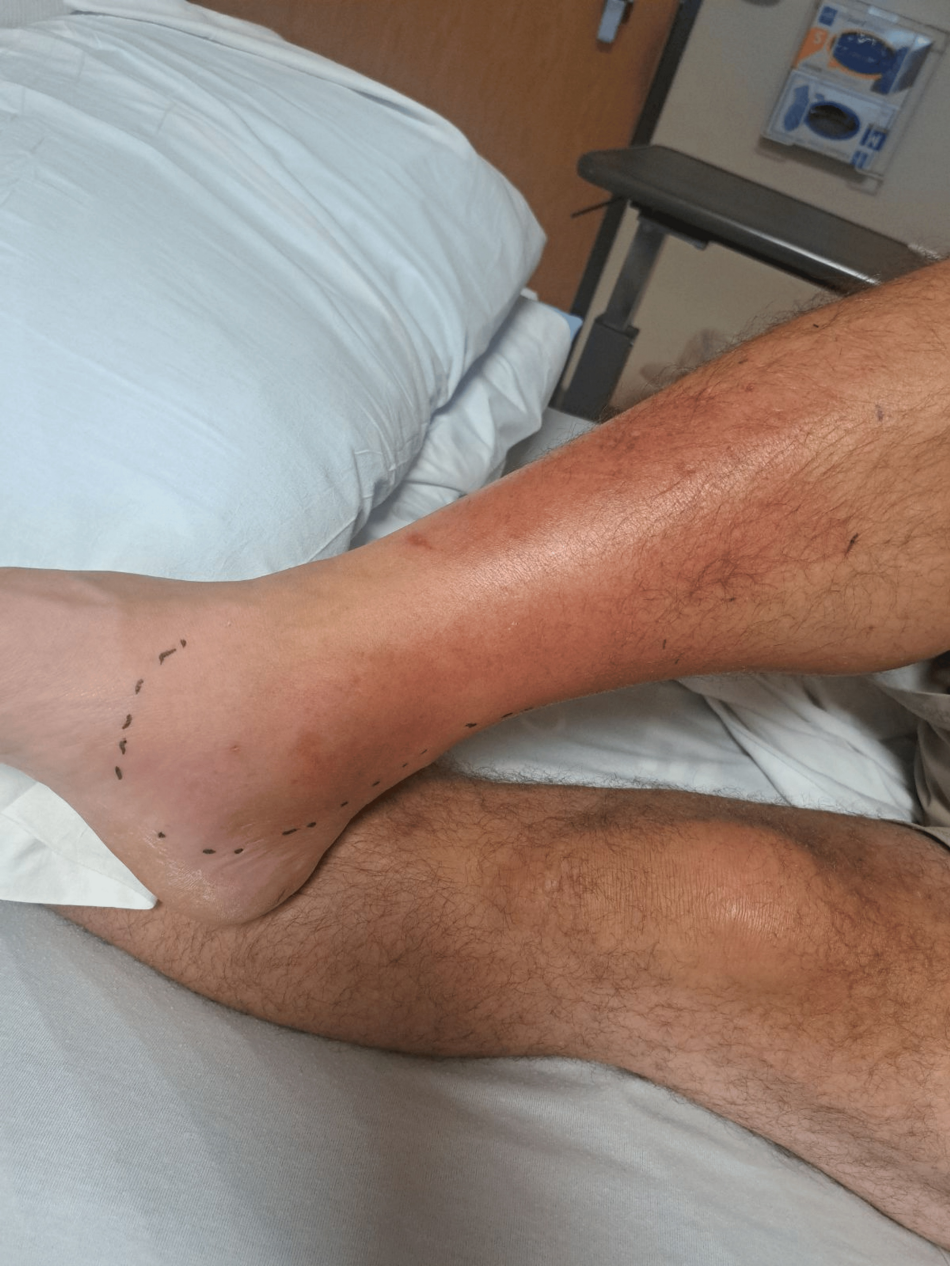
Right lower extremity erythema

A 49-year-old male patient with no comorbidities presented with right lower extremity swelling. The patient stated that he had a fall one week before admission while he was fishing at a freshwater lake that resulted in abrasions to his right knee and calf. He developed progressively worsening swelling in his right lower extremity associated with redness and pain (Figure 1). 

**Figure 2:**
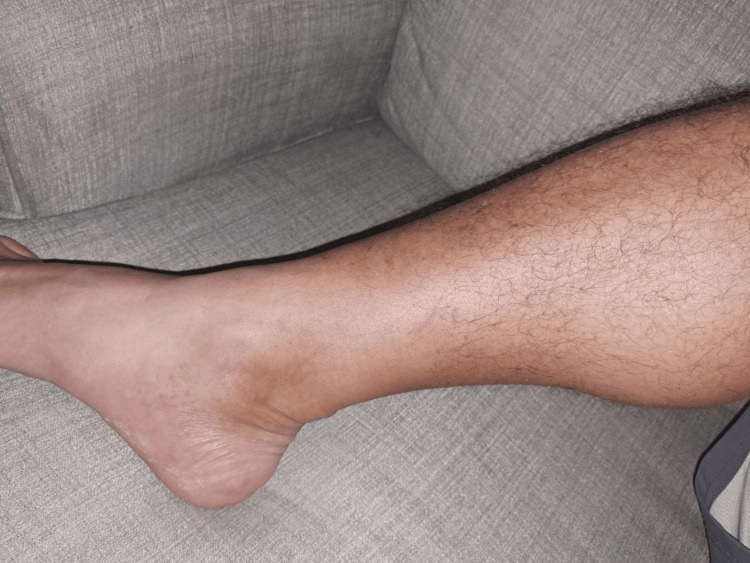
Resolved right lower extremity erythema

He denied cough, abdominal pain, and altered bladder or bowel habits. In the emergency department, the patient was febrile at 100.4°F and tachycardic at 95 beats per minute. The physical exam revealed right lower extremity erythema and swelling with tenderness, but no fluctuance was noted. Lab results revealed leukocytosis of 19,640 per microliter (reference range, 4400-11,000 per microliter) and elevated C-reactive protein at 70.4 (reference range, 0.00-5.00 mg/L). The Doppler ultrasound was negative for deep vein thrombosis. The computed tomography scan of the right lower extremity without contrast showed cellulitis, with no gas in the soft tissues, abscess, and no evidence of osteomyelitis. Blood cultures were positive for *H. jordaniae* (beta-lactamase-producing organism) which was identified by matrix-assisted laser desorption ionization-time of flight mass spectrometry. He was started on oral levofloxacin for two weeks. At a two-week clinic follow-up, his cellulitis was resolved (Figure 2).

## Discussion

*H. jordaniae* is a slow-growing, gram-negative bacillus that is difficult to identify because it is not included in the standard identification databases. Bacteremia caused by *H. jordaniae *has been documented infrequently, and its clinical implications remain poorly understood. *H. Jordaniae* was isolated 14 times in 10 states during 2003-2012 before its identification in 2016 [2]. Identifying* H. jordaniae* in our patient highlights the importance of considering rare pathogens in the differential diagnosis of cellulitis and bacteremia [3]. Accurate microbiological identification, such as 16S rRNA gene sequencing, may be needed sometimes for guiding appropriate antimicrobial therapy, as *H. jordaniae* may exhibit variable susceptibility patterns to commonly used antibiotics [4]. *H. jordaniae* is part of the diverse microflora found in aquatic environments. Infections in humans, particularly bacteremia, have been reported mainly in individuals with underlying malignancies and immunosuppressive states [5,6]. The organism is typically sensitive to tetracycline, fluoroquinolones, and carbapenems, providing a therapeutic window for treatment [6,7]. With the increasing use of advanced molecular techniques for pathogen identification, clinicians should be aware of the evolving infectious diseases, especially those caused by less commonly recognized organisms like *H. jordaniae.*

## Conclusions

This case of *H. jordaniae* bacteremia highlights the importance of considering atypical pathogens presenting with fever and sepsis. Future studies are needed to further clarify the epidemiology, virulence factors, long-term complications, and optimal management strategies for infections caused by *H. jordaniae*.
